# Role of Life’s Essential 8 in the association of night shift work with cardiovascular disease and mortality: a prospective cohort study of UK Biobank participants

**DOI:** 10.7189/jogh.16.04198

**Published:** 2026-08-14

**Authors:** Yeen Huang, Yingping Xiang, Tianzheng Li, Gregory Yoke Hong Lip, Yanhui Gao, Junguo Zhang

**Affiliations:** 1 School of Basic Medical Sciences, Guangzhou University of Chinese Medicine, Guangzhou, China; 2 Occupational Hazard Assessment Institute, Shenzhen Prevention and Treatment Center for Occupational Diseases, Shenzhen, China; 3 Liverpool Centre for Cardiovascular Science at University of Liverpool, Liverpool John Moores University and Liverpool Heart & Chest Hospital, Liverpool, UK; 4 Danish Center for Health Services Research, Department of Clinical Medicine, Aalborg University, Aalborg, Denmark; 5 Department of Cardiology, Lipidology and Internal Medicine with Intensive Coronary Care Unit, Medical University of Bialystok, Bialystok, Poland; 6 Department of Medical Statistics, School of Basic Medicine and Public Health, Jinan University, Guangzhou, China

**Keywords:** night shift work, cardiovascular health, Life’s Essential 8, cardiovascular disease, all-cause mortality, mediation analysis

## Abstract

**Background:**

Night shift work is linked to higher risks of cardiovascular disease (CVD) and mortality, but mechanisms remain unclear. Lifestyle, captured by the American Heart Association’s Life’s Essential 8 (LE8), may act as a modifiable mediator. We aimed to examine the associations of night shift work with CVD and mortality and quantify the mediating role of LE8.

**Methods:**

We analysed 131,844 employed participants without baseline CVD from the UK Biobank (2006–2010). We categorised night shift work as day workers, shift but never/rarely night shifts, some night shifts, and usual/permanent night shifts. LE8 scores included diet, physical activity, nicotine exposure, sleep, body mass index, blood pressure, lipids, and blood glucose. Primary outcomes were incident CVD and all-cause mortality, and secondary outcomes included myocardial infarction (MI), stroke, and atrial fibrillation (AF). We used Cox models to estimate associations and counterfactual mediation analysis to quantify the proportion mediated by LE8.

**Results:**

Over a median follow-up of 13.78 years (interquartile range = 13.09–14.42), 39,115 (29.7%) developed CVD and 5474 (4.2%) died. Compared with day workers, usual/permanent night shifts had higher risks of CVD (hazard ratio (HR) = 1.24; 95% confidence interval (CI) = 1.18–1.31), all-cause mortality (HR = 1.23; 95% CI = 1.07–1.41), AF (HR = 1.31; 95% CI = 1.13–1.51), and MI (HR = 1.29; 95% CI = 1.09–1.51), but not cardiovascular mortality. LE8 mediated 39.2% of the CVD association, 33.1% of all-cause mortality, and 40.8% of MI. Counterfactual analyses suggested that improving cardiovascular health (CVH) could prevent 35.1% of CVD events and 30.2% of deaths among night shift workers.

**Conclusions:**

Night shift work is associated with higher risks of CVD, AF, MI, and all-cause mortality. A substantial proportion of this burden may be preventable through improved CVH. Further studies are needed to evaluate integrated lifestyle interventions in night shift workers.

Shift work, particularly night shift work, is common in modern economies, affects up to 30% of workers in industrialised countries [1], and is especially prevalent in sectors such as healthcare, transport, and services, driven in part by globalisation and the expansion of 24-hour service demands [2]. Night shift work often results in insufficient or misaligned sleep, which has been associated with adverse cardiometabolic effects, including increased risk of cardiovascular disease (CVD) [3]. Pooled evidence from recent meta-analyses indicates that shift work is associated with approximately 17–20% higher risks of CVD morbidity and mortality [1,4]. In a large population-based cohort study, night shift work was associated with a 12% higher risk of all-cause mortality [5]. This has led to an ongoing debate about whether shift work should be classified as an occupational hazard for compensation and how the evidence can be translated into effective policy [6,7].

Several behavioural and physiological pathways have been proposed to explain the link between shift work and CVD, including circadian disruption, autonomic imbalance, systemic inflammation, impaired glucose and lipid metabolism, and a higher prevalence of unhealthy behaviours, such as poor diet, smoking, physical inactivity, and insufficient sleep [8,9]. These factors are central to the Life’s Essential 8 (LE8), an American Heart Association (AHA) measure of cardiovascular health (CVH) comprising four health behaviours (*i.e.* diet, physical activity, sleep, nicotine exposure) and four health factors (*i.e.* body mass index (BMI), blood pressure (BP), blood lipids, blood glucose), validated as a predictor of CVD incidence and mortality [10]. A recent meta-analysis of 34 cohort studies found that higher LE8 scores were associated with approximately 50% lower risks of CVD and CVD mortality [11], and large prospective cohorts have shown similarly reduced risks of all-cause mortality [12].

Although the components of LE8 align closely with the behavioural and physiological pathways linking shift work to CVD, limited research has examined the role of comprehensive lifestyle measures such as LE8 in this association. Specifically, one study demonstrated that LE8 mediates the relationship between chronotype (a circadian phenotype relevant to shift-work tolerance and scheduling [13]) and CVD outcomes, with lifestyle factors such as sleep and physical activity contributing significantly to this mediation [14]. Another study found that higher fibre intake attenuated the elevated coronary heart disease risk associated with night shift work, while meat avoidance was associated with lower risk across work schedules [15]. Together, these findings support the hypothesis that LE8 and its components may lie on relevant pathways linking night shift work to CVD outcomes.

Moreover, most studies have given insufficient attention to the underlying behavioural mechanisms; since shift work is closely linked to unhealthy lifestyles, treating these factors as confounders rather than potential mediators could lead to overadjustment bias [16,17]. In this study, we propose that LE8 captures the cumulative effects of these lifestyle factors and acts as a key mediator in the relationship between night shift work and CVD. The counterfactual framework provides a formal approach to estimating direct and indirect effects under explicit causal assumptions [18].

To address these gaps, we conducted a prospective cohort study using UK Biobank data to examine the association between night shift work and CVD incidence and mortality, to assess the mediating role of LE8 in this association, and to estimate the potential of CVH as quantified by LE8 to reduce the burden of CVD and mortality in night shift workers, using counterfactual analyses.

## METHODS

### Study design and participants

**Figure 1:**
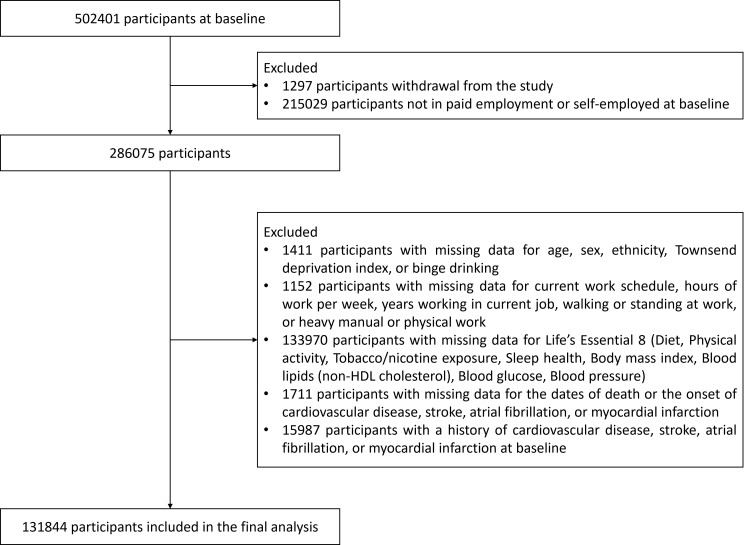
Flowchart for the selection of participants.

The UK Biobank is a population-based prospective cohort of over 500,000 adults aged 40–69 years recruited across the UK between 2006 and 2010. Baseline assessments included questionnaires, physical measurements, biological samples, and interviews [19]. Following the STROBE guideline [20], we included participants who were employed or self-employed at baseline and excluded those not in paid work, with prior CVD, or with missing data on key variables, yielding 131,844 participants for analysis (**Figure 1**). Our analysis aligns with the Journal of Global Health’s Guidelines for Reporting Analyses of Big Data Repositories Open to the Public (Table S1 in the **Online Supplementary Document**) [21].

### Exposure assessment

Night shift work was assessed at baseline among participants in paid employment or self-employment. Participants reported whether their current job involved shift work, defined as work outside 9 am to 5 pm (including evenings, nights, or rotating shifts). Those who reported ‘sometimes, usually, or always’ working shifts were further asked whether their work involved night shifts, defined as working through the normal sleeping hours (*e.g.* 12 am to 6 am) [22,23]. Following previous studies [24,25], participants were classified as day workers, shift but never/rarely night shifts, some night shifts, or usual/permanent night shifts (Tables S2 and S3 in the **Online Supplementary Document**). We considered ‘some night shifts’ and ‘usual/permanent night shifts’ as exposed to night shift work.

### Outcomes

Primary outcomes included CVD events, cardiovascular mortality, and all-cause mortality. The definition of CVD was broad, encompassing a wide range of cardiovascular conditions as defined by the International Classification of Diseases, Tenth Revision codes (I00–I99) (Tables S2 and S4 in the **Online Supplementary Document**) [26,27]. This broad CVD definition reflects the overall burden of CVD in the study population and captures a variety of cardiovascular events.

Secondary outcomes focused on specific subtypes of CVD, including incident stroke (codes I60–I64), atrial fibrillation (AF) (code I48), and myocardial infarction (MI) (codes I21–I23), along with cause-specific mortality from these cardiovascular conditions. These subtypes are more narrowly defined and represent distinct CVD, allowing for a more detailed investigation of the effects of night shift work on specific CVD conditions.

We ascertained the outcomes *via* linkage to primary care data, hospital inpatient records, and death registries. We calculated time to event from baseline to the first occurrence of the outcome, death, loss to follow-up, or the end of follow-up (8 December 2022 in England and 19 December 2022 in Scotland and Wales), whichever came first.

### Measurements of LE8

We used LE8, as defined by the AHA, to assess overall CVH. It includes eight metrics: diet, physical activity, nicotine exposure, sleep health, BMI, blood lipids, blood glucose, and BP. Each metric was scored from 0 to 100, and the overall LE8 score was calculated as the unweighted average of all metrics [10]. In accordance with AHA recommendations, we categorised the participants as having high (80–100), moderate (60–79), or low (0–59) CVH (Table S5 in the **Online Supplementary Document**) [10].

We derived LE8 component scores using established methods recommended by the AHA. The operationalisation of each component is as follows:

1 Diet: We measured diet quality based on a ten-item score reflecting intake of major food groups. Higher scores reflected greater adherence to healthy dietary recommendations, whereas lower scores reflected poorer adherence (Tables S5 and S6 in the **Online Supplementary Document**)
2 Physical activity: We measured physical activity using self-reported data on weekly physical activity, expressed as metabolic equivalent task (MET)-minutes per week. Higher MET-minutes per week corresponded to higher scores.
3 Nicotine exposure: We categorised nicotine exposure by smoking status (*i.e.* current smoker, former smoker, or never smoker) and second-hand smoke exposure. The scoring system assigns lower scores to current smokers and higher scores to non-smokers, with additional consideration for second-hand smoke exposure.
4 Sleep health: We assessed sleep duration based on self-reported average hours of sleep per night. The scoring system gives higher scores for sleep durations within the healthy range, as defined by the AHA.
5 BMI: We calculated BMI from measured height and weight. The scoring system assigns higher scores to individuals with a BMI in the healthy range (<25.0 kg/m^2^) and lower scores to those outside this range.
6 Blood lipids: We used non-high-density lipoprotein cholesterol to assess blood lipids. The scoring criteria assign higher scores to lower non-high-density lipoprotein cholesterol levels (<130 mg/dL) and lower scores to higher levels.
7 Blood glucose: We assessed blood glucose using haemoglobin A1c levels and diabetes history. Lower haemoglobin A1c levels correspond to higher scores.
8 BP: We measured BP as the average of two seated measurements. Higher scores were assigned to individuals with normal BP (systolic < 120 mmHg and diastolic < 80 mmHg).

We obtained antihypertensive and lipid-lowering medication use from linked treatment records and considered it in the scoring of BP and blood lipid metrics.

### Covariates

We selected covariates based on their relevance as potential confounders. Sociodemographic factors included age, sex, race and ethnicity. We assessed socioeconomic status using the Townsend Deprivation Index. We defined binge drinking as consumption of ≥6 standard drinks/d for women and ≥8 standard drinks/d for men [28]. Occupational characteristics included number of work hours per week, years working in the current job, walking or standing at work, and heavy manual or physical labour at work. We collected all covariates at baseline *via* standardised touchscreen questionnaires or verbal interviews.

### Statistical analysis

We summarised baseline characteristics using means (x̄) (standard deviations (SDs)) for continuous variables and proportions for categorical variables, stratified by night shift work status. We evaluated group differences using analysis of variance for continuous variables and a χ^2^ test for categorical variables.

We used Cox proportional hazards regression models to estimate hazard ratios (HRs) and 95% confidence intervals (CIs) for the associations between night shift work and CVD and mortality. We constructed three models: Model 0, adjusted for age and sex; Model 1, additionally adjusted for race and ethnicity, Townsend Deprivation Index, and binge drinking; Model 2, further adjusted for hours of work per week, years working in the current job, walking or standing at work, and heavy manual or physical labour. We assessed the proportional hazards assumption using Schoenfeld residuals and found that it was not violated.

We used causal mediation analysis under the counterfactual framework to estimate the extent to which CVH, measured by the LE8 score, mediated the associations between night shift work and CVD and mortality (Figure S1 in the **Online Supplementary Document**). This approach provides a formal framework for estimating direct and indirect effects under specified causal assumptions [18,29]. We adjusted mediation models for the same covariates as Cox regression Model 2 and estimated the proportion mediated with 95% CIs using 1000 quasi-Bayesian Monte Carlo simulations. We limited the mediation analysis to outcomes significantly associated with night shift work in the primary Cox models.

To assess the potential benefit of improving CVH, we performed counterfactual analyses by hypothetically shifting LE8 scores of night shift workers with low CVH to moderate or high levels, holding other factors constant (Figure S1 in the **Online Supplementary Document**). The difference in outcome risk between observed and counterfactual scenarios reflected the proportion of events potentially preventable through CVH improvement. We derived CIs using 1000 bootstrap replications.

We performed sensitivity analyses to assess the robustness of the findings. First, we excluded participants with events occurring within the first year of follow-up to reduce potential reverse causation. Second, we adjusted for LE8 scores in the Cox models to assess the robustness of the findings and examine the direct effect of LE8 on CVD outcomes, ensuring the associations were not confounded by CVH. This differs from the mediation analysis, where LE8 acts as a mediator in the pathway between night shift work and CVD, estimating both indirect and direct effects [30]. Third, we conducted subgroup analyses by age, sex, race and ethnicity, working hours, and job tenure to explore potential effect modification.

We used SAS, version 9.4 (SAS Institute Inc., Cary, North Carolina, USA) and *R*, version 4.1.2 (R Core Team, Vienna, Austria), for all analyses. All tests were two-sided, and *P* < 0.05 was considered statistically significant. Analyses were conducted from June to August 2025.

## RESULTS

### Baseline characteristics

There were 131,844 employed participants, with an x̄ age of 52.19 (SD = 7.01) years, of whom 50.14% were male (**Table 1**). Among these, 6002 (4.55%) reported some night shifts, and 4498 (3.41%) reported usual/permanent night shifts.

**Table 1:** Baseline characteristics by work schedule*

		Current work schedule				
	**Total (n = 131,844)**	**Day workers (n = 111,186)**	**Shift but never/rarely night shifts (n = 10,158)**	**Some night shifts (n = 6002)**	**Usual/permanent night shifts (n = 4498)**	***P*-value**
**Age in years, x̄ (SD)**	52.19 (7.01)	52.37 (7.02)	51.86 (6.98)	50.56 (6.73)	50.61 (6.66)	<0.001
**Sex**						<0.001
Male	66,108 (50.14)	57,249 (51.49)	5167 (50.87)	2121 (35.34)	1571 (34.93)	
Female	65,736 (49.86)	53,937 (48.51)	4991 (49.13)	3881 (64.66)	2927 (65.07)	
**Race and ethnicity**						<0.001
White	125,088 (94.88)	106,338 (95.64)	9377 (92.32)	5373 (89.52)	4000 (88.93)	
Mixed	898 (0.68)	707 (0.64)	97 (0.95)	61 (1.02)	33 (0.73)	
Asian or Asian British	2421 (1.84)	1765 (1.59)	304 (2.99)	205 (3.42)	147 (3.27)	
Black or Black British	1860 (1.41)	1257 (1.13)	188 (1.85)	220 (3.67)	195 (4.34)	
Chinese	496 (0.37)	385 (0.34)	53 (0.52)	27 (0.44)	31 (0.68)	
Other ethnic group	1081 (0.82)	734 (0.66)	139 (1.37)	116 (1.93)	92 (2.05)	
**Townsend deprivation index, x̄ (SD)**	−1.49 (2.92)	−1.62 (2.85)	−0.80 (3.14)	−0.77 (3.23)	−0.69 (3.18)	<0.001
**Binge drinking**						<0.001
No	128,030 (97.11)	108,175 (97.29)	9785 (96.33)	5721 (95.32)	4349 (96.69)	
Yes	3814 (2.89)	3011 (2.71)	373 (3.67)	281 (4.68)	149 (3.31)	
**Number of work hours a week, x̄ (SD)**	35.49 (12.99)	35.01 (12.84)	35.89 (12.69)	40.26 (13.76)	40.15 (13.87)	<0.001
**Years working in current job, x̄ (SD)**	12.27 (11.46)	12.21 (11.46)	11.52 (11.14)	13.62 (11.82)	13.49 (11.44)	<0.001
**Walking or standing at work**						<0.001
Never or rarely	51,115 (38.77)	47,737 (42.93)	1814 (17.85)	950 (15.83)	614 (13.65)	
Sometimes	41,235 (31.28)	35,202 (31.66)	3105 (30.57)	1896 (31.59)	1032 (22.94)	
Usually	18,210 (13.81)	13,775 (12.39)	2008 (19.77)	1332 (22.19)	1095 (24.34)	
Always	21,284 (16.14)	14,472 (13.02)	3231 (31.81)	1824 (30.39)	1757 (39.07)	
**Heavy manual or physical labour at work**						<0.001
Never or rarely	90,596 (68.72)	83,036 (74.68)	4327 (42.60)	2012 (33.52)	1221 (27.15)	
Sometimes	26,401 (20.02)	18,613 (16.74)	3559 (35.04)	2456 (40.92)	1773 (39.42)	
Usually	7858 (5.96)	5055 (4.55)	1216 (11.97)	801 (13.35)	786 (17.47)	
Always	6989 (5.30)	4482 (4.03)	1056 (10.39)	733 (12.21)	718 (15.96)	
**AHA LE8 Scores (out of 100 possible points), x̄ (SD)**						
Total CVH score	71.09 (11.60)	71.41 (11.57)	70.18 (11.64)	68.80 (11.45)	68.36 (11.59)	<0.001
Dietary recommendations for CVH	61.34 (17.37)	61.41 (17.30)	61.32 (17.59)	60.25 (17.85)	60.97 (17.75)	<0.001
Physical activity	76.89 (36.96)	76.03 (37.36)	80.28 (35.14)	82.66 (33.51)	82.74 (33.53)	<0.001
Tobacco/nicotine exposure	76.66 (32.03)	77.95 (31.23)	71.64 (34.14)	67.90 (36.34)	67.86 (35.90)	<0.001
Sleep health	90.42 (17.12)	91.02 (16.56)	88.73 (18.29)	86.87 (19.79)	84.14 (21.64)	<0.001
BMI	71.35 (27.64)	72.18 (27.37)	68.69 (28.71)	65.63 (28.23)	64.54 (28.67)	<0.001
Blood lipids (non-HDL cholesterol)	49.01 (29.38)	49.16 (29.37)	48.78 (29.39)	47.31 (29.61)	48.12 (29.23)	<0.001
Blood glucose	94.02 (16.06)	94.26 (15.72)	93.15 (17.23)	92.56 (17.80)	91.93 (18.73)	<0.001
BP	49.05 (32.21)	49.27 (32.28)	48.84 (32.07)	47.19 (31.64)	46.57 (31.26)	<0.001
**LE8 score stratification**						<0.001
Low CVH (0–59)	21,902 (16.61)	17,719 (15.93)	1906 (18.76)	1269 (21.14)	1008 (22.41)	
Moderate CVH (60–79)	78,223 (59.33)	65,742 (59.13)	6070 (59.76)	3681 (61.33)	2730 (60.69)	
High CVH (80–100)	31,719 (24.06)	27,725 (24.94)	2182 (21.48)	1052 (17.53)	760 (16.90)	

AHA – American Heart Association, BMI – body mass index, BP – blood pressure, CVH – cardiovascular health, HDL – high-density lipoprotein, LE8 – Life’s Essential 8, SD – standard deviation, x̄ – mean

*Values presented as numbers (%) unless specified otherwise.

Compared with day workers, night shift workers were younger, more likely to be male, from racially and ethnically minoritised groups, and had greater socioeconomic deprivation. They also reported longer weekly work hours, longer job tenure, and greater exposure to physically demanding tasks.

Overall, CVH was lower among night shift workers, with those with some (x̄ = 68.80; SD = 11.45) and usual/permanent (x̄ = 68.36; SD = 11.59) night shifts having lower LE8 scores than day workers (x̄ = 71.41; SD = 11.57). Night shift workers also had lower scores in sleep, BMI, and tobacco exposure, and fewer participants met criteria for high CVH.

### Night shift work, CVD, and mortality

During a median follow-up of 13.78 (interquartile range = 13.09–14.42) years, 39,115 (29.67%) participants developed CVD, 925 (0.70%) died of CVD, and 5474 (4.15%) died of any cause (**Table 2**). In the fully adjusted model (Model 2), usual or permanent night shift work was associated with higher risks of incident CVD (HR = 1.24; 95% CI = 1.18–1.31; *P* < 0.001) and all-cause mortality (HR = 1.23; 95% CI = 1.07–1.41; *P* < 0.01) compared with day workers. There were no associations between night shift work and cardiovascular mortality after multivariable adjustment. Participants working some night shifts also showed an elevated risk of CVD (HR = 1.11; 95% CI = 1.06–1.17; *P* < 0.001).

**Table 2:** Associations of night shift work with CVD, cardiovascular mortality, and all-cause mortality

		Model 0*		Model 1†		Model 2‡	
	**Cases, n/N (%)**	**HR (95% CI)**	***P*-value**	**HR (95% CI)**	***P*-value**	**HR (95% CI)**	***P*-value**
**CVD**							
Day workers	32,283/111,186 (29.04)	ref	NA	ref	NA	ref	NA
Shift but never/rarely night shifts	3,339/10,158 (32.87)	1.21 (1.17–1.25)	<0.001	1.18 (1.13–1.22)	<0.001	1.12 (1.08–1.17)	<0.001
Some night shifts	1,918/6002 (31.96)	1.22 (1.16–1.27)	<0.001	1.17 (1.12–1.22)	<0.001	1.11 (1.06–1.17)	<0.001
Usual/permanent night shifts	1,575/4498 (35.02)	1.37 (1.30–1.44)	<0.001	1.32 (1.25–1.39)	<0.001	1.24 (1.18–1.31)	<0.001
**Cardiovascular mortality**							
Day workers	771/111,186 (0.69)	ref	NA	ref	NA	ref	NA
Shift but never/rarely night shifts	71/10,158 (0.70)	1.07 (0.84–1.36)	0.59	1.01 (0.79–1.29)	0.94	0.94 (0.74–1.21)	0.65
Some night shifts	48/6002 (0.80)	1.25 (0.93–1.67)	0.14	1.17 (0.87–1.56)	0.31	1.11 (0.82–1.49)	0.51
Usual/permanent night shifts	35/4498 (0.78)	1.22 (0.87–1.72)	0.25	1.15 (0.82–1.61)	0.43	1.07 (0.76–1.52)	0.69
**All-cause mortality**							
Day workers	4,554/111,186 (4.10)	ref	NA	ref	NA	ref	NA
Shift but never/rarely night shifts	453/10,158 (4.46)	1.15 (1.04–1.26)	<0.01	1.11 (1.01–1.22)	<0.05	1.06 (0.96–1.17)	0.26
Some night shifts	250/6002 (4.17)	1.16 (1.02–1.32)	<0.05	1.11 (0.98–1.27)	0.10	1.05 (0.92–1.20)	0.45
Usual/permanent night shifts	217/4498 (4.82)	1.34 (1.17–1.54)	<0.001	1.31 (1.14–1.50)	<0.001	1.23 (1.07–1.41)	<0.01

CI – confidence interval, CVD – cardiovascular disease, HR – hazard ratio, NA – not available, ref – reference

*Model 0 adjusted for age and sex.

†Model 1 additionally adjusted for race and ethnicity, Townsend deprivation index, and binge drinking.

‡Model 2 additionally adjusted for hours of work per week, years working in current job, walking or standing at work, and heavy manual or physical work.

In analyses of secondary outcomes, usual or permanent night shift work was associated with increased risks of AF (HR = 1.31; 95% CI = 1.13–1.51; *P* < 0.001) and MI (HR = 1.29; 95% CI = 1.09–1.51; *P* < 0.01), but not stroke or cause-specific cardiovascular mortality (Table S7 in the **Online Supplementary Document**).

### Mediation effects of LE8

Among participants exposed to night shift work, CVH assessed using the LE8 score mediated a substantial proportion of the associations with cardiovascular outcomes (**Table 3**). LE8 explained 52.0% (95% CI = 30.3–72.9) of the association between some night shift work and incident CVD and 39.2% (95% CI = 29.3–50.0) of the association for usual or permanent night shift work. For all-cause mortality, LE8 mediated 33.1% (95% CI = 15.3–57.5) of the association for usual or permanent night shift work. We did not perform a mediation analysis for cardiovascular mortality due to a lack of significant total associations.

**Table 3:** Mediation proportion of the association between night shift work and CVD, cardiovascular mortality, and all-cause mortality attributed to LE8

	LE8	
	% mediated (95% CI)*	***P*-value**
**CVD**		
Day workers	NA	NA
Shift but never/rarely night shifts	28.3 (18.9–40.1)	<0.001
Some night shifts	52.0 (30.3–72.9)	<0.001
Usual/permanent night shifts	39.2 (29.3–50.0)	<0.001
**Cardiovascular mortality**		
Day workers	NA	NA
Shift but never/rarely night shifts†	NA	NA
Some night shifts†	NA	NA
Usual/permanent night shifts†	NA	NA
**All-cause mortality**		
Day workers	NA	NA
Shift but never/rarely night shifts†	NA	NA
Some night shifts†	NA	NA
Usual/permanent night shifts	33.1 (15.3–57.5)	<0.001

CI – confidence interval, CVD – cardiovascular disease, NA – not available

*Adjusted for age, sex, race and ethnicity, Townsend Deprivation Index, binge drinking, hours of work per week, years working in current job, walking or standing at work, and heavy manual or physical work.

†We did not perform a mediation analysis since there was no significant association with night shift work in the primary Cox model.

In analyses of secondary outcomes, LE8 mediated 17.5% (95% CI = 9.2–30.8) of the association between usual or permanent night shift work and AF and 40.8% (95% CI = 18.5–67.8) of the association with MI (Table S8 in the **Online Supplementary Document**). We did not perform mediation analyses for stroke or cause-specific cardiovascular mortality due to nonsignificant total associations.

### Counterfactual estimates of preventable CVD and mortality

**Figure 2:**
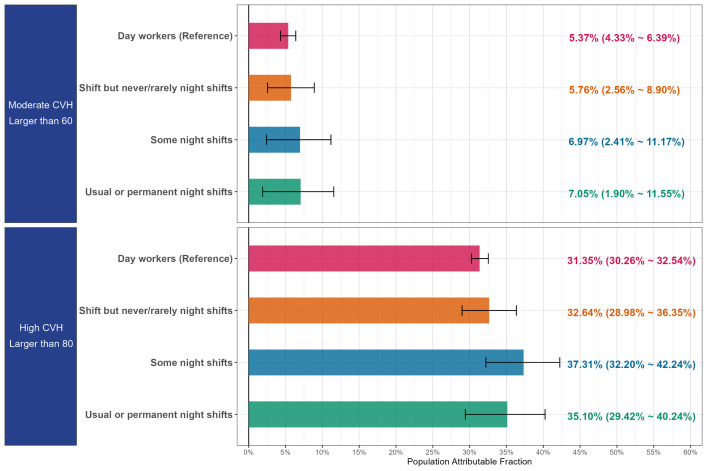
Counterfactual estimates of preventable CVD events by level of CVH. CVD – cardiovascular disease, CVH – cardiovascular health.

We used counterfactual analyses to estimate the proportion of CVD and all-cause mortality events that could be prevented if CVH were improved to ideal levels (**Figure 2**). If CVH improved to high levels, 37.31% (95% CI = 32.20–42.24) of CVD events among participants with some night shifts and 35.10% (95% CI = 29.42–40.24) among those with usual or permanent night shifts were estimated to be preventable. Even if CVH improved only to moderate levels, 6.97% (95% CI = 2.41–11.17) and 7.05% (95% CI = 1.90–11.55) of CVD events were estimated to be preventable in these groups, respectively.

For all-cause mortality, analysis was limited to participants with usual or permanent night shifts, among whom 30.20% (95% CI = 12.62–45.53) of deaths were estimated to be preventable under a scenario of high CVH.

### Subgroup and sensitivity analyses

Significant effect modification of the association with CVD incidence was observed by age, sex, and weekly working hours (*P* for interaction <0.05). In particular, the association between usual or permanent night shift work and CVD was stronger among participants working ≥55 hours per week (HR = 1.52; 95% CI = 1.31–1.78; *P* < 0.001) compared with those working <55 hours (HR = 1.25; 95% CI = 1.18–1.32; *P* < 0.001). Similar trends were observed among younger and female participants. No significant interactions were observed for cardiovascular mortality (Figures S2 and S3 in the **Online Supplementary Document**).

For all-cause mortality, a significant interaction was observed for weekly working hours (*P* for interaction <0.01), with stronger associations for some night shift work among participants working ≥55 hours per week (HR = 1.66; 95% CI = 1.21–2.29; *P* < 0.01) compared with those working <55 hours per week (HR = 0.97; 95% CI = 0.84–1.12; *P* = 0.67) (Figure S4 in the **Online Supplementary Document**).

Sensitivity analyses, excluding participants who developed outcomes within the first year of follow-up, yielded similar associations between night shift work and both CVD and all-cause mortality. In a separate model, additional adjustment for LE8 scores modestly attenuated these associations, but did not materially change the findings, supporting the robustness of the results (Table S9 in the **Online Supplementary Document**).

## DISCUSSION

Among the 131,844 employed participants, we found that night shift work was associated with increased risks of CVD and all-cause mortality. Night shift workers had lower LE8 scores, and fewer met the criteria for high CVH. Mediation analyses indicated that LE8 explained a substantial proportion of the observed associations, particularly for incident MI. Counterfactual analyses suggested that improving CVH could prevent approximately one-third of CVD events and all-cause deaths among night shift workers.

Our findings are consistent with prior research and confirm the association between night shift work and CVD. A meta-analysis of 173,010 participants and two UK Biobank studies reported similar associations between shift work and CVD outcomes [1,2,5]. Our results, including associations between night shift work and increased risks of MI and AF, align with previous studies, though there was no association with stroke except in individuals with genetic susceptibility [25,31,32]. Although we found a significant association with CVD incidence, the lack of association with cardiovascular mortality may be due to several factors, including the low number of cardiovascular deaths, competing risks from other causes, and survival bias, where healthier individuals are more likely to remain employed and in follow-up. However, the categorisation of night shift exposure into four groups may obscure heterogeneity within the ‘some night shifts’ category. More precise quantification of exposure, such as the amount of time spent on night shifts or the cumulative duration of night shift work, would strengthen dose-response interpretation and provide a clearer understanding of the relationship between night shift work and CVD outcomes.

In our subgroup analyses, we observed that longer working hours enhanced the association between night shift work and CVD incidence, aligning with previous studies linking cumulative fatigue and overtime stress to adverse health outcomes [33]. Extended work hours contribute to fatigue and psychological stress, disrupting circadian rhythms and increasing physiological strain, which can elevate cardiovascular risk factors such as BP and inflammation [34]. Additionally, we found significant sex-specific differences in the relationship between night shift work and CVD risk. Women may be more vulnerable to these risks due to hormonal fluctuations, such as the disruption of oestrogen’s protective effects [35]. Furthermore, women often face higher psychosocial stress and caregiving responsibilities, which can worsen lifestyle factors like sleep, diet, and physical activity [36]. These factors, combined with occupational segregation, where women often experience lower job control, may further increase CVD risk. Future research integrating these factors could provide a more comprehensive understanding of how working hours, sex, and night shift work interact to affect CVD risk.

Conventional adjustment for lifestyle factors may lead to overadjustment bias, especially for chronic diseases like CVD, where unhealthy behaviours often act as mediators [16]. To date, only two studies have examined the mediating role of lifestyle in the association between shift work and health, each focusing on a single behaviour [2,23]. Given that unhealthy behaviours often co-occur, such approaches may underestimate their overall impact. Using LE8, a composite measure of CVH from the AHA, we comprehensively assessed the mediating role of lifestyle and found that LE8 explained a substantial proportion of the associations between night shift work and cardiovascular outcomes, particularly MI. These findings highlight the value of integrated lifestyle metrics in clarifying behavioural and physiological pathways linking shift work to CVD.

Our findings suggest that improving CVH could reduce a significant proportion of CVD events and all-cause mortality among night shift workers. However, the effectiveness of interventions to improve CVH in this population still requires further investigation. While integrated lifestyle interventions targeting factors such as diet, physical activity, and sleep may hold promise for reducing cardiovascular risks, more research is needed to evaluate their feasibility and long-term effectiveness among night shift workers. Qualitative studies have shown that single-focus interventions, such as sleep education, light therapy, or melatonin, have had limited or inconsistent effectiveness in mitigating health risks associated with shift work [37,38]. Future strategies may involve integrating behavioural, cognitive, and emotional self-regulation techniques to help manage fatigue and reduce health risks [39]. However, further research is necessary to determine their practical applicability and effectiveness in shift-working populations.

This study has several strengths. We applied a counterfactual framework to quantify the potential benefit of improving CVH among night shift workers, providing actionable estimates of preventable disease burden. We used LE8, a validated composite metric, which enabled an integrated assessment of lifestyle, addressing limitations of prior studies focused on individual behaviours. In addition, we examined both overall and specific cardiovascular outcomes, offering more detailed insights into the health risks of night shift work. However, we acknowledge that these counterfactual prevention estimates assume ideal shifts to LE8 levels without considering practical factors such as feasibility, adherence, and occupational constraints. These factors may lead to an overestimation of the preventable burden. While the estimates are useful for policy, future research should account for these real-world limitations to provide a more accurate assessment of CVH improvements in night shift workers.

This study also has limitations. First, despite extensive covariate adjustment, residual confounding from unmeasured factors (*e.g.* genetic susceptibility, accessibility of medical services, and stress factors, including occupational, psychosocial, and environmental stressors associated with shift work) cannot be excluded. Second, we assessed shift work exposure only at baseline, which limits our ability to capture the cumulative or time-varying effects of shift work. Although our method of assessing night shift exposure is consistent with that used in most prior studies [2,15,23], it may still introduce exposure misclassification bias. Third, the study population was limited to employed individuals aged 40–69 years, which may reduce the generalisability of our findings to younger shift workers with potentially different cardiometabolic trajectories. Including younger populations in future research would help determine whether the observed associations apply to them and how cardiovascular risks vary across age groups. Lastly, we did not account for potential differences in healthcare access between shift and day workers, which may influence the diagnosis and reporting of cardiovascular events. The available data lack detailed information on healthcare utilisation, which may have resulted in underreporting or misclassification of outcomes.

### Policy implications

We found that LE8 mediated part of the observed associations between night shift work and cardiovascular outcomes. Because LE8 captures modifiable factors such as sleep, diet, and physical activity, it may provide a useful framework for evaluating workplace health interventions. These modifiable factors suggest that improving CVH may help reduce cardiovascular risk among night shift workers. Worksite wellness programmes, which aim to improve these lifestyle factors (*e.g.* enhancing sleep hygiene and promoting physical activity), could help reduce cardiovascular risk [40]. Additionally, our study aligns with the Total Worker Health framework, which combines workplace safety and health promotion to improve overall well-being [41]. Total Worker Health focuses on addressing both work-related hazards and health behaviours, making it a valuable approach for improving CVH in night shift workers. Future research could explore the effectiveness of such interventions, using LE8 as a framework to assess their impact on CVH among night shift workers.

## CONCLUSIONS

Night shift work was associated with higher risks of CVD and overall mortality. A substantial proportion of this burden may be preventable through improvements in overall cardiovascular health. Further research is needed to evaluate the effectiveness of integrated lifestyle interventions aimed at reducing cardiovascular risk in night shift workers.

## Additional material


Online Supplementary Document

## Data Availability

**Data availability:** The data used in this study are available to approved researchers through the UK Biobank website (http://www.ukbiobank.ac.uk/). Restrictions apply to their availability, and this study accessed the data under application number 90162.
