# Supplementary material for: Role of Life’s Essential 8 in the association of night shift work with cardiovascular disease and mortality: a prospective cohort study of UK Biobank participants
Source: J Glob Health. 2026 Aug 14;16:04198. doi: 10.7189/jogh.16.04198 (PMC13474059; doi:10.7189/jogh.16.04198)
Supplement: Supplementary file 1 [file jogh-16-04198-s001.pdf]

Supplement to: Huang Y, Xiang Y, Li T, Lip G, Gao Y, Zhang J. Role of Life's Essential 8 in the association of night shift work with cardiovascular disease andmortality: a prospective cohort study of UK Biobank participants. J Glob Health. 2026;16:04198.

Table S1. Outline of JoGH's Guidelines for Reporting Analyses of Big Data Repositories Open to the Public (GRABDROP) items.
Table S2. Selected Variables from the UK Biobank and their Coding
Table S3. Classification Criteria for Current Work Schedule
Table S4. Ascertainment for Cardiovascular Events in the UK Biobank Study
Table S5. Scoring Criteria and Classification Categories for Life's Essential 8 Metrics
Table S6. Components of Contemporary Dietary Recommendations for Cardiovascular Health in the UK Biobank
Table S7. Associations of Night Shift Work with Stroke, Atrial Fibrillation, Myocardial Infarction, and Cause-Specific Mortality
Table S8. Mediation Proportion of the Association Between Night Shift Work and Stroke, Atrial Fibrillation, and Myocardial Infarction Incidence and Mortality Attributed to Life's Essential 8
Table S9. Sensitive Analyses of Association Between Night Shift Work and Cardiovascular Disease and Mortality
Figure S1. Directed Acyclic Graph Illustrating the Causal Pathway Between Night Shift Work and Cardiovascular Outcomes, with Life's Essential 8 as a Mediator
Figure S2. Associations Between Night Shift Work and Cardiovascular Disease by Subgroup
Figure S3. Associations Between Night Shift Work and Cardiovascular Mortality by Subgroup
Figure S4. Associations Between Night Shift Work and All-Cause Mortality by Subgroup

Table S1. Adherence to JoGH's Guidelines for Reporting Analyses of Big Data Repositories Open to the Public (GRABDROP) items.

**JoGH guideline items**
1. Please list all papers published by each co-author in previous 3 years that were based on secondary analysis of a big data repository

In this study, the following co-authors have published papers based on secondary analysis of large data repositories:

1. Huang Y, Wu R, He J, Xiang Y. Evaluating ChatGPT-4.0's data analytic proficiency in epidemiological studies: A comparative analysis with SAS, SPSS, and R. *J Glob Health*. 2024;14:04070. doi: 10.7189/jogh.14.04070
2. Huang Y, Xiang Y, Zhou W, Li G, Zhao C, Zhang D, Fang S. Long working hours and all-cause mortality in China: A 26-year follow-up study. *Scand J Work Environ Health*. 2023;49(8):539-548. doi: 10.5271/sjweh.4115
3. Huang Y, Xiang Y, Zhou W, Li G, Zhao C, Zhang D, Fang S. Short working hours and perceived stress: Findings from a population-based nationwide study. *Heliyon*. 2023;9(11):e21919. doi: 10.1016/j.heliyon.2023.e21919
4. Zhang J, Wei S, Chen G, Lip GYH, Lin H. Life-course exposure to PM2.5 components and genetic susceptibility on the risk of atrial fibrillation and subsequent heart failure. *Eur J Prev Cardiol*. 2025;zwaf751. doi: 10.1093/eurjpc/zwaf751
5. Zhang J, Chen G, Wei S, et al. Impact of air pollution and multimorbidity on the risk of incident atrial fibrillation. *Heart*. 2025;19 Jun; doi: 10.1136/heartjnl-2024-325451
6. Zhang J, Wei S, Chen G, Zhang B, Wang Y, Huang ZH, Lip GYH, Wang X. The role of Life's Essential 8 and multimorbidity in the risk of new-onset atrial fibrillation: observations from a large prospective cohort study. *Int J Surg*. 2025;111(10):6885-6893. doi: 10.1097/JS9.0000000000002831
7. Zhang J, Luo L, Chen G, et al. Associations of ambient air pollution with incidence and dynamic progression of atrial fibrillation. *Sci Total Environ*. 2024;951:175710. doi: 10.1016/j.scitotenv.2024.175710
8. Zhang J, Chen G, Habudele Z, Wang X, Cai M, Li H, Gao Y, Lip GYH, Lin H. Relation of Life's Essential 8 to the genetic predisposition for cardiovascular outcomes and all-cause mortality: results from a national prospective cohort. *Eur J Prev Cardiol*. 2023;30(15):1676-1685. doi: 10.1093/eurjpc/zwad179
9. Zhang J, Chen G, Xia H, Wang X, Wang C, Cai M, Gao Y, Lip GYH, Lin H. Associations of Life's Essential 8 and fine particulate matter pollution with the incidence of atrial fibrillation. *J Hazard Mater*. 2023;459:132114. doi: 10.1016/j.jhazmat.2023.132114
10. Zhang J, Chen G, Wang C, et al. Associations of risk factor burden and genetic predisposition with the 10-year risk of atrial fibrillation: observations from a large prospective study of 348,904 participants. *BMC Med*. 2023;21:88. doi: 10.1186/s12916-023-02798-7

2. Please explain the key elements of your study design and the use of the available datasets that make your study an original scientific contribution

This study used data from the UK Biobank, a large, well-established cohort, to investigate the association between night shift work and cardiovascular disease (CVD), while assessing the mediating role of Life's Essential 8 (LE8), a composite measure of cardiovascular health. We focused on 131,844 employed participants without CVD at baseline, classifying them by their night shift work exposure (day workers, shift but never/rarely night shifts, some night shifts, and usual/permanent night shifts).

The study design leverages the UK Biobank's extensive dataset, including detailed health, lifestyle, and demographic information, which allowed us to apply advanced statistical methods, including Cox models and counterfactual mediation analysis, to examine the direct and indirect effects of LE8 on the relationship between night shift work and CVD outcomes. Our findings provide novel insights into how improving cardiovascular health could reduce the CVD burden among night shift workers.

By utilizing a large, publicly accessible dataset and integrating comprehensive lifestyle measures, this study presents an original contribution to understanding the health risks of night shift work and highlights the potential of lifestyle interventions to mitigate these risks.

3. Please list all publications that addressed similar research questions in the same dataset and indicate where you cited them in your paper

Our study utilized data from the UK Biobank, and we cited the one relevant publication that addressed similar research questions:

1. Ho FK, Celis-Morales C, Gray SR, Demou E, Mackay D, Welsh P, Katikireddi SV, Sattar N, Pell JP: Association and pathways between shift work and cardiovascular disease: a prospective cohort study of 238,661 participants from UK Biobank. *Int J Epidemiol*. 2022;51(2):579-590. [Cited as reference 2 in our paper]

- This study explored the association between shift work and CVD outcomes within the UK Biobank cohort but did not examine the mediating role of lifestyle factors such as LE8. Unlike their study, our research comprehensively investigates the relationship between night shift work and multiple outcomes, including CVD incidence, all-cause mortality, and CVD mortality. Additionally, we examine the mediating effect of LE8 in these associations, providing deeper insights into how cardiovascular health can influence the risks linked to night shift work.

4. Please explain how you addressed multiple testing through an appropriately rigorous statistical threshold and indicate this in the methods section

Given the multiple outcomes assessed in our study, including CVD incidence, all-cause mortality, CVD mortality, myocardial infarction (MI), stroke, and atrial fibrillation (AF), we treated these outcomes as independent hypotheses and maintained a significance threshold of $P<0.05$ for all tests. While we did not apply a formal multiple testing correction, we addressed the potential risk of false-positive results by conducting rigorous subgroup and sensitivity analyses. These additional tests consistently supported our main findings, suggesting that the observed associations are robust.

5. Please declare to what extent have AI chatbots been used in developing your paper and to which parts of the paper did they contribute

AI chatbots were used to assist with language editing and refinement to ensure that the manuscript adhered to standard English expressions. This assistance was primarily focused on improving the clarity and flow of the text. Apart from this language support, no AI chatbots were used in other parts of the paper.

Table S2. Selected Variables from the UK Biobank and their Coding

Data-Field	Group	Data-Field code
**Sample restriction: Employment status**	Include only: "In paid employment or self-employed"	6142
**Exposures**		
**1. Current work schedule (Primary exposure)**	Combined variable (4 categories):<br>• Day workers (reference group)<br>• Shift but never or rarely night shifts<br>• Some night shifts<br>• Usual or permanent night shifts	See Table S3
**2.Life's Essential 8 (LE8) (Mediator) (Dietary recommendations, physical activity, tobacco/nicotine exposure, sleep health, BMI, blood lipids, blood glucose, blood pressure)**	Tertiles:<br>• High CVH (80-100 points)<br>• Moderate CVH (60-79 points)<br>• Low CVH (0-59 points)	See Table S5 and S6
**Outcomes**		
**1.Cardiovascular Disease**		
**Time-to-event variable: Date outcome first reported**	-	Category 2409
**Cardiovascular events**	No/Yes	131270-131423
**Stroke**	No/Yes	131360-131369
**Atrial fibrillation**	No/Yes	131350-131351
**Myocardial infarction**	No/Yes	131298-131303
**2.Mortality**		
**Time-to-event variables: Date of death**	-	40000
**All-cause mortality**	No/Yes	40001
**Cardiovascular mortality**	No/Yes	40001
**Stroke mortality**	No/Yes	40001
**Atrial fibrillation mortality**	No/Yes	40001
**Myocardial infarction mortality**	No/Yes	40001
**Covariates**		
**Age**	mean (SD)	21022
**Sex**	Female/Male	31
**Race and ethnicity**	White/Mixed/Asian or Asian British/Black or Black British/Chinese/Other ethnic group	21000
**Townsend Deprivation index**	mean (SD)	22189
**Binge drinking**	No/Yes	1558
**No. of work hours a week**	mean (SD)	767
**Years working in current job**	mean (SD)	757
**Walking or standing at work**	Never or rarely/Sometimes/Usually/Always	806
**Heavy manual or physical labor at work**	Never or rarely/Sometimes/Usually/Always	816
**Sensitivity analyses/Subgroup analyses**		
**Age**	≤50/>50	21022
**Sex**	Female/Male	31
**Race and ethnicity**	White/Non-white	21000
**Length of working week for main job**	<55/≥55	767
**Time employed in main current job**	≤12/>12	757

Table S3. Classification Criteria for Current Work Schedule

Shift work status (Data-Field code: 826)	Night shift work schedule (Data-Field code: 3426)	Combined variable: Current work schedule
**Never/rarely**	-	Day workers
**Sometimes**	Never/rarely	Shift but never or rarely night shifts
	Sometime	Some night shifts
	Usually	Usual or permanent night shifts
	Always	Usual or permanent night shifts
**Usually**	Never/rarely	Shift but never or rarely night shifts
	Sometime	Some night shifts
	Usually	Usual or permanent night shifts
	Always	Usual or permanent night shifts
**Always**	Never/rarely	Shift but never or rarely night shifts
	Sometime	Some night shifts
	Usually	Usual or permanent night shifts
	Always	Usual or permanent night shifts

Table S4. Ascertainment for Cardiovascular Events in the UK Biobank Study

Events	ICD-10
**Cardiovascular disease**	I00-I99
**Stroke**	I60-I64
**Atrial fibrillation**	I48
**Myocardial infarction**	I21-I23

Table S5. Scoring Criteria and Classification Categories for Life's Essential 8 Metrics

LE8 metric	Method of measurement	Quantification of LE8 metric	Score and classification
**Diet**	Measurement: A more recent definition of ideal intake of dietary components for cardiovascular health	Scoring:<br>Points diets score (points)<br>100 8–10<br>80 6–7<br>50 4–5<br>25 2–3<br>0 0–1	The LE8 score is scaled from 0 to 100 points, calculated as the unweighted average of all 8 component metric scores.<br><br>In the present study, overall LE8 scores of 80 to 100 are considered High CVH; 60 to 79, Moderate CVH; and 0 to 59 points, low CVH.
**Physical activity (PA)**	Self-reported minutes of moderate or vigorous PA per week	Scoring:<br>Points Minutes<br>100 ≥150<br>90 120–149<br>80 90–119<br>60 60–89<br>40 30–59<br>20 1–29<br>0 0	
**Tobacco/nicotine exposure**	Self-reported use of cigarettes; or secondhand smoke exposure	Scoring:<br>Points Status<br>100 Never smoker<br>75 Former smoker, quit ≥5 y<br>50 Former smoker, quit 1–<5 y<br>25 Former smoker, quit <1 y<br>0 Current smoker<br>Subtract 20 points (unless score is 0) for living with active indoor smoker in home	
**Sleep health**	Self-reported average hours of sleep per night	Scoring:<br>Points Level<br>100 7–<9<br>90 9–<10<br>70 6–<7<br>40 5–<6 or ≥10<br>20 4–<5<br>0 <4	

**Body mass index**	Measurement: Body weight (kilograms) divided by height squared (meters squared)	Scoring:<br>Points Level<br>100 <25<br>70 25.0–29.9<br>30 30.0–34.9<br>15 35.0–39.9<br>0 ≥40.0	
**Blood lipids (non-HDL cholesterol)**	Measurement: Plasma total and HDL cholesterol with calculation of non–HDL cholesterol	Metric: Non–HDL cholesterol (mg/dL)<br>Scoring:<br>Points Level<br>100 <130<br>60 130–159<br>40 160–189<br>20 190–219<br>0 ≥220<br>If drug-treated level, subtract 20 points	
**Blood glucose**	Measurement: HbA1c and history of Diabetes	Metric: HbA1c (%)<br>Scoring:<br>Points Level<br>100 No history of diabetes HbA1c <5.7<br>60 No diabetes and HbA1c 5.7–6.4 (prediabetes)<br>40 Diabetes with HbA1c <7.0<br>30 Diabetes with HbA1c 7.0–7.9<br>20 Diabetes with HbA1c 8.0–8.9<br>10 Diabetes with Hb A1c 9.0–9.9<br>0 Diabetes with HbA1c ≥10.0	
**Blood pressure (BP)**	Measurement: Appropriately measured systolic and diastolic blood pressures	Metric: Systolic and diastolic BPs (mmHg)<br>Scoring:<br>Points Level<br>100 <120/<80 (optimal)<br>75 120–129/<80 (elevated)<br>50 130–139 or 80–89 (stage 1 hypertension)<br>25 140–159 or 90–99<br>0 ≥160 or ≥100<br>Subtract 20 points if treated level	

Table S6. Components of Contemporary Dietary Recommendations for Cardiovascular Health in the UK Biobank

Diet component	Intake goal	Field IDs	Amount per serving
**Fruit**	3 servings/day	1309 (pieces fresh fruit/day)<br>1319 (pieces dried fruit/day)	1309 – 1 piece<br>1319 – 5 pieces
**Vegetable**	3 servings/day	1289 (tablespoons cooked vegetables/day)<br>1299 (salad/raw vegetables/day)	3 heaped tablespoons
**Whole grains**	3 servings/day	1438, 1448 (whole meal/wholegrain bread slices/week)<br>1458, 1468 (bran/oat/muesli cereal bowls/week)	1438/1448 – 1 slice/day<br>1458/1468 – 1 bowl/day
**(Shell)fish**	≥2 servings/week	1329 (oily fish/week)<br>1339 (non-oily fish/week)	Once/week
**Dairy**	2 servings/day	1408 (cheese/week)<br>1418 (milk type)	1408 – 1 piece/day<br>1418 – 1 glass/day if consumption of any type of milk
**Vegetable oils**	2 servings/day	1428 (Flora Pro-Active/Benecol spread)<br>2654 (Flora Pro-Active/Benecol, soft margarine -, olive oil based -, polyunsaturated/sunflower oil based -, other low/reduced fat spread)<br>1438 (bread slices/week)	1 serving/day if in combination with eating at least 2 slices of bread (ID 1438)
**Refined grains**	≤2 servings/day	1438, 1448 (white, brown, other bread slices/week)<br>1458, 1468 (biscuit, other cereals/week)	1438/1448 – 1 slice/day<br>1458/1468 – 1 bowl/day
**Processed meats**	≤1 serving/week	1349 (processed meat/week or daily)<br>3680 (age when last ate meat)	1349 – 1 piece/day<br>3680 – 0 pieces/day if indicated having never eaten meat
**Unprocessed meats**	≤2 servings/day	1359 (poultry/week or day)<br>1369 (beef/week or day)<br>1379 (lamb or mutton/week or day)<br>1389 (pork/week or day)<br>3680 (age when last ate meat)	1359-1389 – once/week<br>3680 – 0 pieces/day if indicated having never eaten meat
**Sugar-sweetened beverages**	Don't drink	6144 (never consumes drinks containing sugar)	Only 0 servings were possible here.

Field IDs and serving sizes for each dietary component were derived from the general baseline questionnaire in the UK Biobank. Participants were considered to have adequate intake if they met the specified intake goals. Dietary components were scored as 1 if the goal was met and 0 if not, yielding a total diet score ranging from 0 to 10.

Table S7. Associations of Night Shift Work with Stroke, Atrial Fibrillation, Myocardial Infarction, and Cause-Specific Mortality

		Model 0[a]		Model 1[b]		Model 2[c]	
**Characteristics**	**Cases (%)**	**HR (95% CI)**	***P* value**	**HR (95% CI)**	***P* value**	**HR (95% CI)**	***P* value**
**Stroke**							
Day workers	1772/111186 (1.59)	1 [Reference]	NA	1 [Reference]	NA	1 [Reference]	NA
Shift but never/rarely night shifts	163/10158 (1.60)	1.06 (0.90-1.24)	0.50	1.02 (0.87-1.20)	0.77	0.97 (0.82-1.14)	0.67
Some night shifts	99/6002 (1.65)	1.14 (0.93-1.39)	0.21	1.09 (0.89-1.34)	0.40	1.03 (0.83-1.26)	0.80
Usual/permanent night shifts	86/4498 (1.91)	1.33 (1.07-1.65)	<0.05	1.28 (1.03-1.59)	<0.05	1.18 (0.95-1.48)	0.14
**AF**							
Day workers	4212/111186 (3.79)	1 [Reference]	NA	1 [Reference]	NA	1 [Reference]	NA
Shift but never/rarely night shifts	400/10158 (3.94)	1.11 (1.00-1.23)	0.05	1.10 (0.99-1.22)	0.07	1.08 (0.97-1.20)	0.17
Some night shifts	225/6002 (3.75)	1.10 (0.96-1.26)	0.17	1.10 (0.96-1.25)	0.18	1.08 (0.94-1.24)	0.29
Usual/permanent night shifts	199/4498 (4.42)	1.32 (1.14-1.52)	<0.001	1.33 (1.15-1.54)	<0.001	1.31 (1.13-1.51)	<0.001
**MI**							
Day workers	2700/111186 (2.43)	1 [Reference]	NA	1 [Reference]	NA	1 [Reference]	NA
Shift but never/rarely night shifts	289/10158 (2.85)	1.23 (1.09-1.38)	<0.01	1.18 (1.05-1.33)	<0.01	1.10 (0.97-1.25)	0.13
Some night shifts	180/6002 (3.00)	1.21 (1.04-1.41)	<0.05	1.17 (1.00-1.36)	0.05	1.08 (0.93-1.26)	0.31
Usual/permanent night shifts	160/4498 (3.56)	1.45 (1.24-1.70)	<0.001	1.41 (1.20-1.65)	<0.001	1.29 (1.09-1.51)	<0.01
**Storke mortality**							
Day workers	137/111186 (0.12)	1 [Reference]	NA	1 [Reference]	NA	1 [Reference]	NA
Shift but never/rarely night shifts	12/10158 (0.12)	1.01 (0.56-1.83)	0.97	0.97 (0.53-1.74)	0.91	0.86 (0.47-1.58)	0.63
Some night shifts	5/6002 (0.08)	0.90 (0.37-2.20)	0.82	0.86 (0.35-2.10)	0.73	0.80 (0.32-1.99)	0.63
Usual/permanent night shifts	4/4498 (0.09)	0.95 (0.35-2.58)	0.92	0.90 (0.33-2.46)	0.84	0.86 (0.31-2.36)	0.76
**AF mortality**							
Day workers	85/111186 (0.08)	1 [Reference]	NA	1 [Reference]	NA	1 [Reference]	NA
Shift but never/rarely night shifts	8/10158 (0.08)	1.15 (0.56-2.37)	0.71	1.11 (0.54-2.30)	0.78	1.03 (0.49-2.16)	0.94
Some night shifts	3/6002 (0.05)	0.83 (0.26-2.62)	0.75	0.78 (0.25-2.49)	0.68	0.77 (0.24-2.47)	0.66
Usual/permanent night shifts	4/4498 (0.09)	1.59 (0.58-4.35)	0.37	1.59 (0.58-4.37)	0.37	1.51 (0.54-4.22)	0.43
**MI mortality**							
Day workers	170/111186 (0.15)	1 [Reference]	NA	1 [Reference]	NA	1 [Reference]	NA
Shift but never/rarely night shifts	17/10158 (0.17)	1.16 (0.70-1.90)	0.57	1.09 (0.66-1.80)	0.73	1.03 (0.62-1.72)	0.91
Some night shifts	16/6002 (0.27)	1.75 (1.04-2.92)	<0.05	1.63 (0.97-2.74)	0.06	1.59 (0.93-2.70)	0.09
Usual/permanent night shifts	7/4498 (0.16)	1.03 (0.48-2.19)	0.95	0.97 (0.45-2.07)	0.93	0.93 (0.43-2.01)	0.85

Abbreviations: HR, hazard ratio; CI, confidence interval; AF, atrial fibrillation; MI, myocardial infarction; NA, not available.

[a]Model 0 adjusted for age and sex.

[b]Model 1 additionally adjusted for race and ethnicity, Townsend deprivation index, and binge drinking.

[c]Model 2 adjusted for terms in model 1 and hours of work per week, years working in current job, walking or standing at work, and heavy manual or physical work.

Table S8. Mediation Proportion of the Association Between Night Shift Work and Stroke, Atrial Fibrillation, and Myocardial Infarction Incidence and Mortality Attributed to Life's Essential 8

Characteristics	Life's Essential 8	
	% Mediated (95% CI)[a]	*P* value
**Stroke**		
Day workers	NA	NA
Shift but never/rarely night shifts[b]	Ø	Ø
Some night shifts[b]	Ø	Ø
Usual/permanent night shifts[b]	Ø	Ø
**AF**		
Day workers	NA	NA
Shift but never/rarely night shifts[b]	Ø	Ø
Some night shifts[b]	Ø	Ø
Usual/permanent night shifts	17.5 (9.2-30.8)	<0.001
**MI**		
Day workers	NA	NA
Shift but never/rarely night shifts[b]	Ø	Ø
Some night shifts[b]	Ø	Ø
Usual/permanent night shifts	40.8 (18.5-67.8)	<0.001
**Stroke mortality**		
Day workers	NA	NA
Shift but never/rarely night shifts[b]	Ø	Ø
Some night shifts[b]	Ø	Ø
Usual/permanent night shifts[b]	Ø	Ø
**AF mortality**		
Day workers	NA	NA
Shift but never/rarely night shifts[b]	Ø	Ø
Some night shifts[b]	Ø	Ø
Usual/permanent night shifts[b]	Ø	Ø
**MI mortality**		
Day workers	NA	NA
Shift but never/rarely night shifts[b]	Ø	Ø
Some night shifts[b]	Ø	Ø
Usual/permanent night shifts[b]	Ø	Ø

Abbreviations: CI, confidence interval; AF, atrial fibrillation; MI, myocardial infarction; NA, not available; Ø, no significant association.

[a]Adjusted for age, sex, race and ethnicity, Townsend deprivation index, binge drinking, hours of work per week, years working in current job, walking or standing at work, and heavy manual or physical work.

[b]While the *P* value of hazard ratio was large than 0.05, mediation proportion was not calculated.

Table S9. Sensitive Analyses of Association Between Night Shift Work and Cardiovascular Disease and Mortality

	CVD		Cardiovascular mortality		All-cause mortality	
**Characteristics**	**HR (95% CI)[a]**	***P* value**	**HR (95% CI)[a]**	***P* value**	**HR (95% CI)[a]**	***P* value**
**Sensitivity analysis 1[b]**						
**Current work schedule**						
Day workers	1 [Reference]	NA	1 [Reference]	NA	1 [Reference]	NA
Shift but never/rarely night shifts	1.09 (1.05-1.13)	<0.001	0.90 (0.70-1.16)	0.43	1.04 (0.94-1.15)	0.41
Some night shifts	1.06 (1.01-1.12)	<0.05	1.06 (0.79-1.43)	0.70	1.02 (0.89-1.16)	0.78
Usual/permanent night shifts	1.17 (1.11-1.24)	<0.001	1.01 (0.72-1.43)	0.94	1.18 (1.03-1.36)	<0.05
**Sensitivity analysis 2[c]**						
**Current work schedule**						
Day workers	1 [Reference]	NA	1 [Reference]	NA	1 [Reference]	NA
Shift but never/rarely night shifts	1.09 (1.05-1.13)	<0.001	0.91 (0.71-1.16)	0.44	1.04 (0.94-1.14)	0.49
Some night shifts	1.06 (1.01-1.11)	<0.05	1.03 (0.77-1.39)	0.83	1.01 (0.89-1.16)	0.83
Usual/permanent night shifts	1.17 (1.11-1.23)	<0.001	0.98 (0.70-1.39)	0.93	1.17 (1.02-1.34)	<0.05

Abbreviations: HR, hazard ratio; CI, confidence interval; CVD, cardiovascular disease; NA, not available.

[a]Adjusted for age, sex, race and ethnicity, Townsend deprivation index, binge drinking, hours of work per week, years working in current job, walking or standing at work, and heavy manual or physical work.

[b]In sensitivity analysis 1, participants with events occurring within the first year of follow-up were excluded to reduce potential reverse causation.

[c]In sensitivity analysis 2, Cox models were additionally adjusted for LE8 scores to assess whether CVH attenuated the associations.

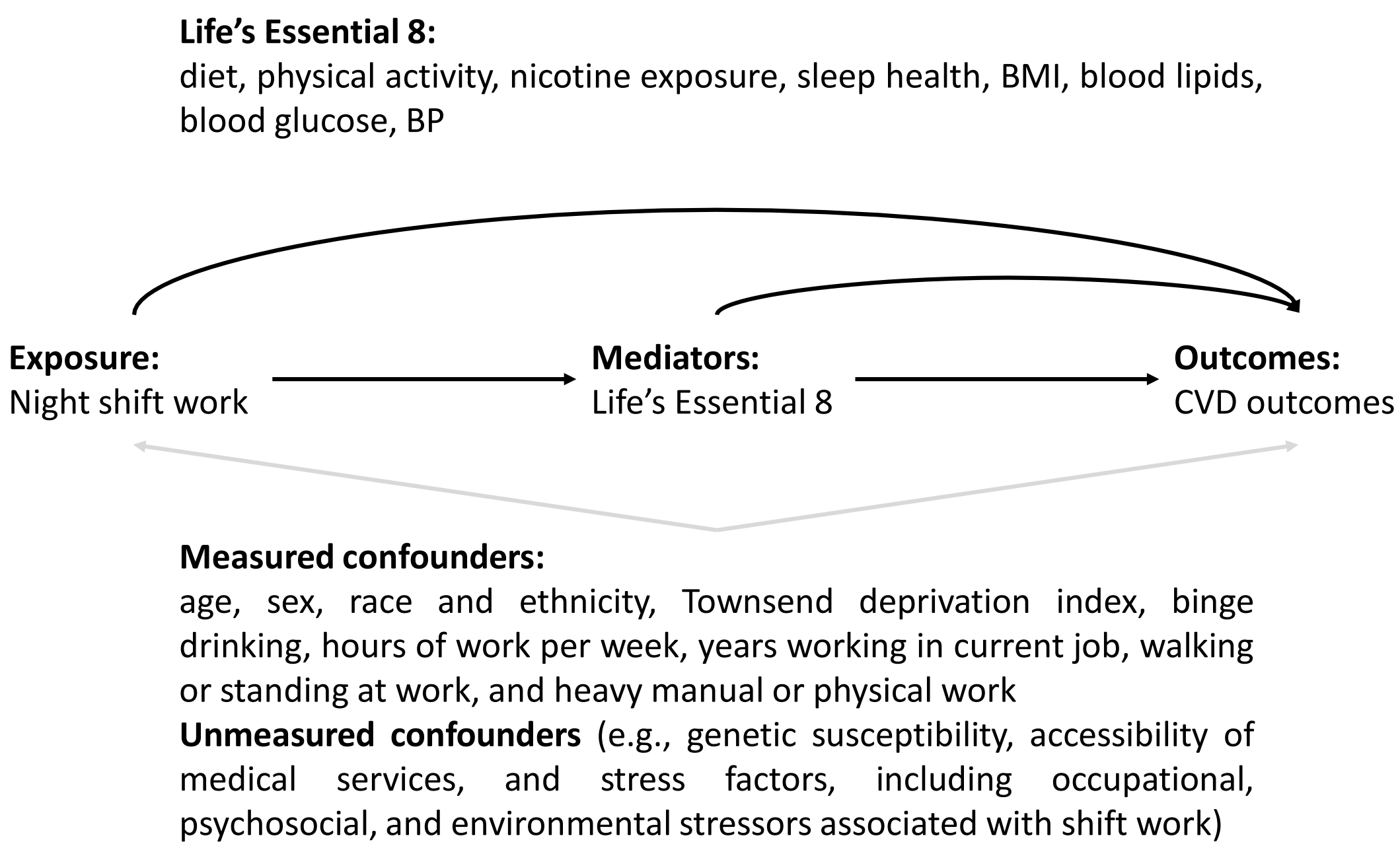

Figure S1. Directed Acyclic Graph Illustrating the Causal Pathway Between Night Shift Work and Cardiovascular Outcomes, with Life’s Essential 8 as a Mediator

This Directed Acyclic Graph (DAG) represents the proposed causal structure in our study, illustrating the relationship between night shift work and cardiovascular disease (CVD) outcomes, with Life’s Essential 8 (LE8) acting as a mediator. The graph shows that night shift work influences CVD outcomes both directly and indirectly, through lifestyle factors captured by LE8, including diet, physical activity, sleep health, and other behavioral factors. The DAG also highlights the potential confounding factors (e.g., age, sex, socioeconomic status) that could simultaneously affect both night shift work and CVD outcomes, and thus need to be controlled for in our analysis. Importantly, the diagram justifies the classification of LE8 as a mediator rather than a confounder, as it captures the cumulative effect of lifestyle behaviors influenced by night shift work, which subsequently affect CVD risk. This causal structure is foundational to our counterfactual mediation analysis, which estimates both the direct and indirect effects of night shift work on CVD outcomes via LE8. Abbreviations: DAG, directed acyclic graph; LE8, Life’s Essential 8; CVD, cardiovascular disease; BMI, body mass index; BP, blood pressure.

Subgroup Analysis: Cardiovascular Disease Incidence

Subgroup	Category	Work schedule	Events/n	HR (95% CI)	P-value	P-interaction
Age	≤50 years	Day workers	8667/46483	1.00 (Reference)	Reference	0.015
		Shift but never/rarely night shifts	1075/4616	1.24 (1.16-1.32)	<0.001	
		Some night shifts	761/3198	1.20 (1.11-1.29)	<0.001	
		Usual or permanent night shifts	630/2384	1.36 (1.25-1.47)	<0.001	
	>50 years	Day workers	23616/64703	1.00 (Reference)	Reference	
		Shift but never/rarely night shifts	2264/5542	1.14 (1.09-1.19)	<0.001	
		Some night shifts	1157/2804	1.10 (1.04-1.17)	0.002	
		Usual or permanent night shifts	945/2114	1.24 (1.16-1.32)	<0.001	
Ethnicity	Other ethnic groups	Day workers	1416/4848	1.00 (Reference)	Reference	0.132
		Shift but never/rarely night shifts	268/781	1.13 (0.99-1.29)	0.060	
		Some night shifts	213/629	1.10 (0.95-1.27)	0.199	
		Usual or permanent night shifts	159/498	1.07 (0.91-1.27)	0.403	
	White	Day workers	30867/106338	1.00 (Reference)	Reference	
		Shift but never/rarely night shifts	3071/9377	1.17 (1.13-1.22)	<0.001	
		Some night shifts	1705/5373	1.14 (1.08-1.20)	<0.001	
		Usual or permanent night shifts	1416/4000	1.30 (1.23-1.37)	<0.001	
No. of work hours a week	<55 hours/week	Day workers	30775/106207	1.00 (Reference)	Reference	0.027
		Shift but never/rarely night shifts	3128/9569	1.17 (1.12-1.21)	<0.001	
		Some night shifts	1700/5315	1.14 (1.08-1.20)	<0.001	
		Usual or permanent night shifts	1382/4046	1.25 (1.18-1.32)	<0.001	
	≥55 hours/week	Day workers	1508/4979	1.00 (Reference)	Reference	
		Shift but never/rarely night shifts	211/589	1.23 (1.07-1.42)	0.005	
		Some night shifts	218/687	1.08 (0.93-1.25)	0.297	
		Usual or permanent night shifts	193/452	1.52 (1.31-1.78)	<0.001	
Years working in current job	≤12 years	Day workers	18405/65886	1.00 (Reference)	Reference	0.066
		Shift but never/rarely night shifts	2027/6225	1.19 (1.14-1.25)	<0.001	
		Some night shifts	1033/3187	1.20 (1.13-1.28)	<0.001	
		Usual or permanent night shifts	847/2389	1.32 (1.23-1.42)	<0.001	
	>12 years	Day workers	13878/45300	1.00 (Reference)	Reference	
		Shift but never/rarely night shifts	1312/3933	1.14 (1.08-1.21)	<0.001	
		Some night shifts	885/2815	1.06 (0.99-1.13)	0.106	
		Usual or permanent night shifts	728/2109	1.23 (1.14-1.32)	<0.001	
Sex	Female	Day workers	13919/57249	1.00 (Reference)	Reference	0.027
		Shift but never/rarely night shifts	1494/5167	1.22 (1.16-1.29)	<0.001	
		Some night shifts	576/2121	1.18 (1.09-1.28)	<0.001	
		Usual or permanent night shifts	474/1571	1.32 (1.21-1.45)	<0.001	
	Male	Day workers	18364/53937	1.00 (Reference)	Reference	
		Shift but never/rarely night shifts	1845/4991	1.13 (1.08-1.19)	<0.001	
		Some night shifts	1342/3881	1.12 (1.06-1.18)	<0.001	
		Usual or permanent night shifts	1101/2927	1.26 (1.19-1.34)	<0.001	

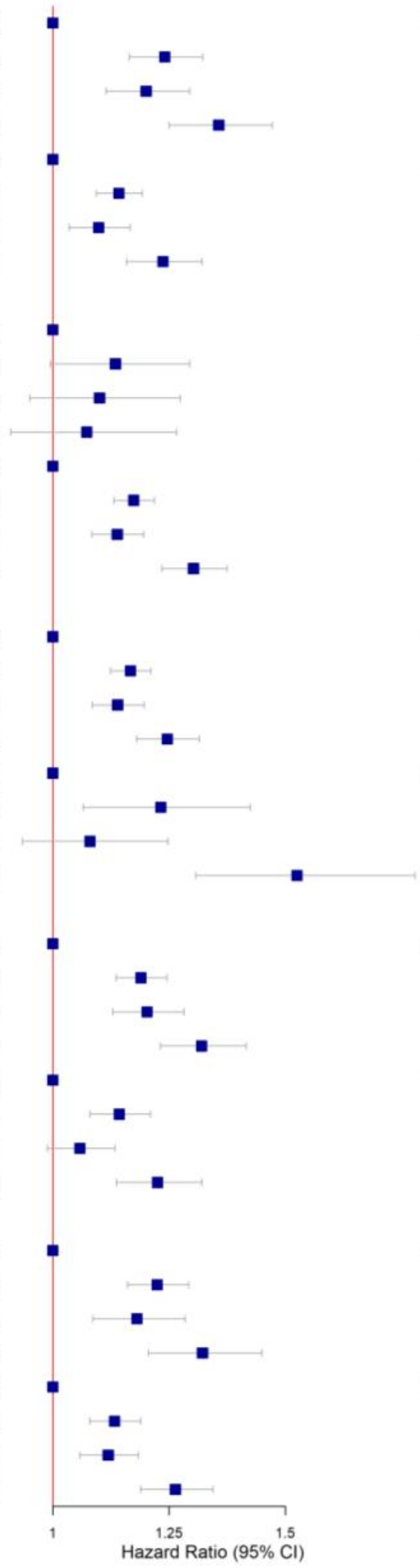

Figure S2. Associations Between Night Shift Work and Cardiovascular Disease by Subgroup

Adjusted for age, sex, race and ethnicity, Townsend deprivation index, binge drinking, hours of work per week, years working in current job, walking or standing at work, and heavy manual or physical work. Each group adjusted for the other covariates except itself. Work hours were categorized as <55 or ≥55 hours/week (long working hours). Years working in current job were categorized by the median (≤12 vs >12 years). Abbreviations: HR, hazard ratio; CI, confidence interval.

Subgroup Analysis: Cardiovascular Mortality

Subgroup	Category	Work schedule	Events/n	HR (95% CI)	P-value	P-interaction
Age	≤50 years	Day workers	145/46483	1.00 (Reference)	Reference	0.558
		Shift but never/rarely night shifts	17/4616	1.06 (0.64-1.76)	0.810	
		Some night shifts	18/3198	1.39 (0.84-2.28)	0.197	
		Usual or permanent night shifts	10/2384	1.04 (0.55-1.99)	0.902	
	>50 years	Day workers	626/64703	1.00 (Reference)	Reference	
		Shift but never/rarely night shifts	54/5542	0.99 (0.75-1.30)	0.921	
		Some night shifts	30/2804	0.99 (0.68-1.43)	0.938	
		Usual or permanent night shifts	25/2114	1.11 (0.74-1.66)	0.607	
Ethnicity	Other ethnic groups	Day workers	22/4848	1.00 (Reference)	Reference	0.023
		Shift but never/rarely night shifts	4/781	0.96 (0.33-2.82)	0.947	
		Some night shifts	8/629	2.12 (0.92-4.89)	0.076	
		Usual or permanent night shifts	0/498	0.00 (<0.10->20)	0.996	
	White	Day workers	749/106338	1.00 (Reference)	Reference	
		Shift but never/rarely night shifts	67/9377	1.01 (0.78-1.29)	0.965	
		Some night shifts	40/5373	1.02 (0.74-1.40)	0.927	
		Usual or permanent night shifts	35/4000	1.19 (0.85-1.68)	0.306	
No. of work hours a week	<55 hours/week	Day workers	734/106207	1.00 (Reference)	Reference	0.094
		Shift but never/rarely night shifts	67/9569	1.01 (0.79-1.30)	0.908	
		Some night shifts	36/5315	0.95 (0.68-1.33)	0.776	
		Usual or permanent night shifts	30/4046	1.06 (0.73-1.53)	0.754	
	≥55 hours/week	Day workers	37/4979	1.00 (Reference)	Reference	
		Shift but never/rarely night shifts	4/589	0.88 (0.31-2.48)	0.808	
		Some night shifts	12/687	2.42 (1.23-4.74)	0.010	
		Usual or permanent night shifts	5/452	1.42 (0.54-3.69)	0.475	
Years working in current job	≤12 years	Day workers	459/65886	1.00 (Reference)	Reference	0.135
		Shift but never/rarely night shifts	52/6225	1.16 (0.87-1.55)	0.312	
		Some night shifts	22/3187	0.94 (0.61-1.44)	0.766	
		Usual or permanent night shifts	17/2389	0.93 (0.57-1.51)	0.754	
	>12 years	Day workers	312/45300	1.00 (Reference)	Reference	
		Shift but never/rarely night shifts	19/3933	0.72 (0.45-1.15)	0.171	
		Some night shifts	26/2815	1.30 (0.87-1.95)	0.202	
		Usual or permanent night shifts	18/2109	1.29 (0.80-2.07)	0.302	
Sex	Female	Day workers	201/57249	1.00 (Reference)	Reference	0.116
		Shift but never/rarely night shifts	28/5167	1.51 (1.02-2.25)	0.041	
		Some night shifts	8/2121	1.15 (0.57-2.34)	0.698	
		Usual or permanent night shifts	5/1571	0.94 (0.39-2.29)	0.892	
	Male	Day workers	570/53937	1.00 (Reference)	Reference	
		Shift but never/rarely night shifts	43/4991	0.82 (0.60-1.12)	0.221	
		Some night shifts	40/3881	1.10 (0.80-1.52)	0.565	
		Usual or permanent night shifts	30/2927	1.12 (0.77-1.62)	0.543	

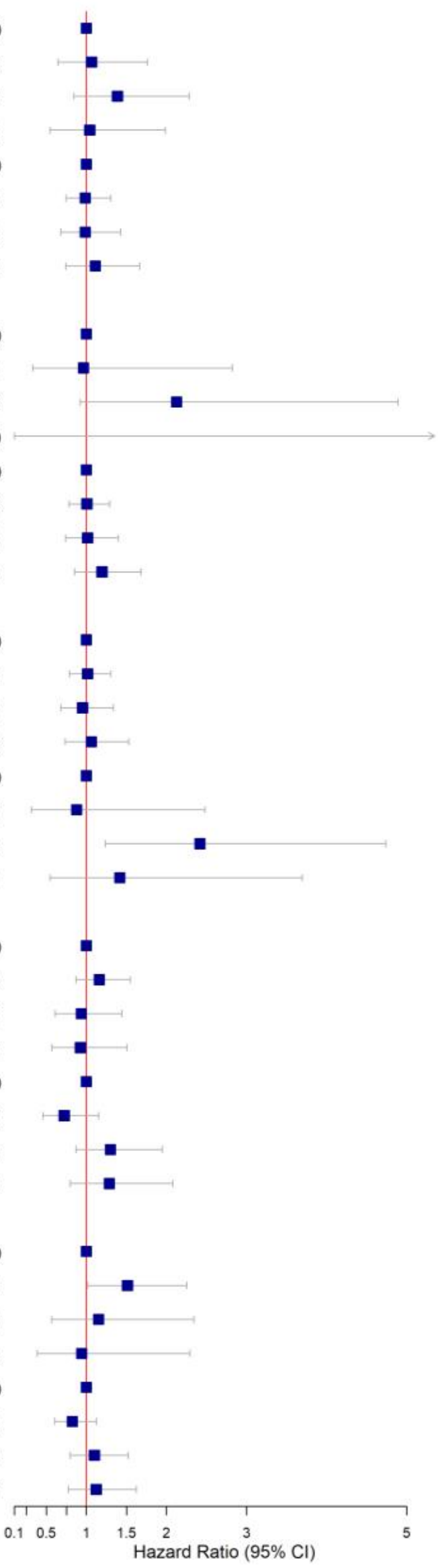

Figure S3. Associations Between Night Shift Work and Cardiovascular Mortality by Subgroup

Adjusted for age, sex, race and ethnicity, Townsend deprivation index, binge drinking, hours of work per week, years working in current job, walking or standing at work, and heavy manual or physical work. Each group adjusted for the other covariates except itself. Work hours were categorized as <55 or ≥55 hours/week (long working hours). Years working in current job were categorized by the median (≤12 vs >12 years). Abbreviations: HR, hazard ratio; CI, confidence interval.

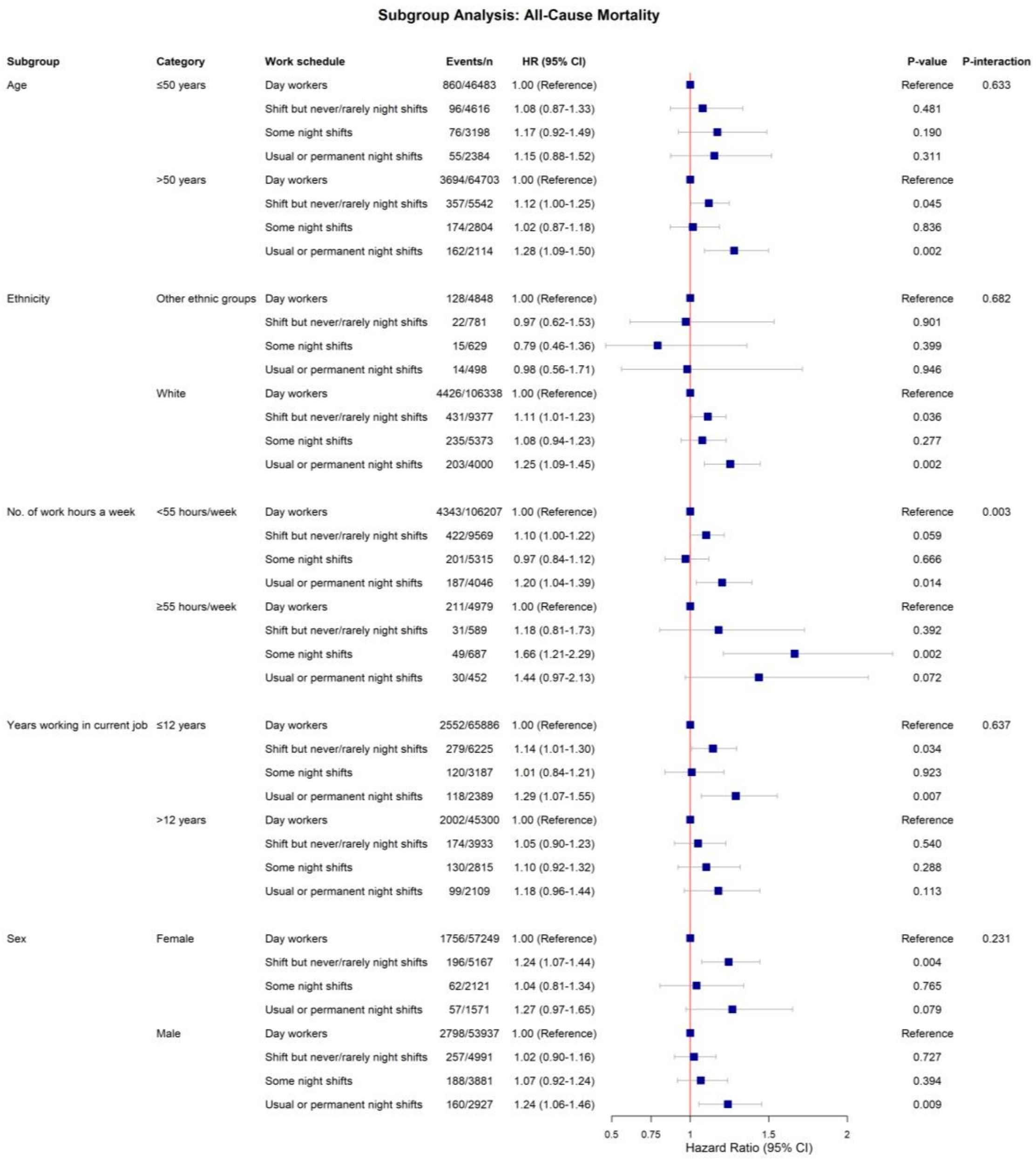

Figure S4. Associations Between Night Shift Work and All-Cause Mortality by Subgroup

Adjusted for age, sex, race and ethnicity, Townsend deprivation index, binge drinking, hours of work per week, years working in current job, walking or standing at work, and heavy manual or physical work. Each group adjusted for the other covariates except itself. Work hours were categorized as <55 or ≥55 hours/week (long working hours). Years working in current job were categorized by the median (≤12 vs >12 years). Abbreviations: HR, hazard ratio; CI, confidence interval.
